# Machine Learning Descriptors for CO_2_ Capture Materials

**DOI:** 10.3390/molecules30030650

**Published:** 2025-02-01

**Authors:** Ibrahim B. Orhan, Yuankai Zhao, Ravichandar Babarao, Aaron W. Thornton, Tu C. Le

**Affiliations:** 1 School of Science, STEM College, RMIT University, G.P.O. Box 2476, Melbourne, VIC 3001, Australia; 2 School of Engineering, STEM College, RMIT University, G.P.O. Box 2476, Melbourne, VIC 3001, Australia; s3924784@student.rmit.edu.au; 3 CSIRO Manufacturing Flagship, Clayton, Melbourne, VIC 3168, Australia

**Keywords:** CO_2_ capture, machine learning, featurization, descriptors

## Abstract

The influence of machine learning (ML) on scientific domains continues to grow, and the number of publications at the intersection of ML, CO_2_ capture, and material science is growing rapidly. Approaches for building ML models vary in both objectives and the methods through which materials are represented (i.e., featurised). Featurisation based on descriptors, being a crucial step in building ML models, is the focus of this review. Metal organic frameworks, ionic liquids, and other materials are discussed in this paper with a focus on the descriptors used in the representation of CO_2_-capturing materials. It is shown that operating conditions must be included in ML models in which multiple temperatures and/or pressures are used. Material descriptors can be used to differentiate the CO_2_ capture candidates through descriptors falling under the broad categories of charge and orbital, thermodynamic, structural, and chemical composition-based descriptors. Depending on the application, dataset, and ML model used, these descriptors carry varying degrees of importance in the predictions made. Design strategies can then be derived based on a selection of important features. Overall, this review predicts that ML will play an even greater role in future innovations in CO_2_ capture.

## 1. Introduction

Since the 1800s, the effects of anthropogenic CO_2_ on the climate have been discussed [1]. With current atmospheric levels surpassing 424 ppm as of November 2024, the Earth is experiencing concentrations comparable to those between 4.1 and 4.5 million years ago [2]. To combat this drastic change in CO_2_ concentration, net CO_2_ emissions must be reduced. A promising route for reducing atmospheric CO_2_ emissions in the near term is carbon capture and storage (CCS) and carbon dioxide removal (CDR). CCS technologies have been proposed to meet emission reduction targets of up to 90% from fixed-point sources [3]. CDR aims to remove carbon dioxide directly from the atmosphere using biological approaches, such as enhanced mineralisation, biomass storage, soil carbon enhancement, and reforestation, or engineering approaches, such as direct air capture (DAC). However, many attempts to commercialise CCS and ensure its widespread adoption have failed [4]. Discovering better adsorbents that enhance the performance of CCS and reduce costs is necessary to achieve wider adoption.

In a 2023 review [5], Dziejarski et al. compiled a list of important properties when evaluating materials for CO_2_ capture [6,7]. These include equilibrium adsorption capacity, CO_2_ selectivity, adsorption kinetics, mechanical properties, the chemical nature of the surface, pore characteristics, chemical and thermal stability, regeneration capacity, stability in adsorption/desorption cycles, production costs, and environmental implications. These properties were all influential on a candidate material being selected for CCS. While pre-combustion, post-combustion, and oxy-combustion offer different conditions under which CO_2_ is captured, typically, post-combustion or direct air capture conditions are considered. Solid sorbents, compared with liquids, are brought to the forefront in their paper as they have garnered significant attention and are considered among the most promising materials for CCS [8].

Among the most widely used solid materials for CO_2_ capture, with strengths and drawbacks in each of the aforementioned criteria, are zeolites, metal oxides, silica, metal-organic frameworks (MOFs), and carbon materials such as carbon nanotubes and activated carbon. Despite significant research having been conducted on these materials, the working adsorption capacity, cycle lifetime, and multi-cycle durability are the key properties that require improvement before these solid sorbents become economical [9]. Computational methods can focus on predicting these aspects of materials. For example, current trends indicate computational methods are frequently being applied to evaluate the selectivity and adsorption capacities of these materials.

**Figure 1:**
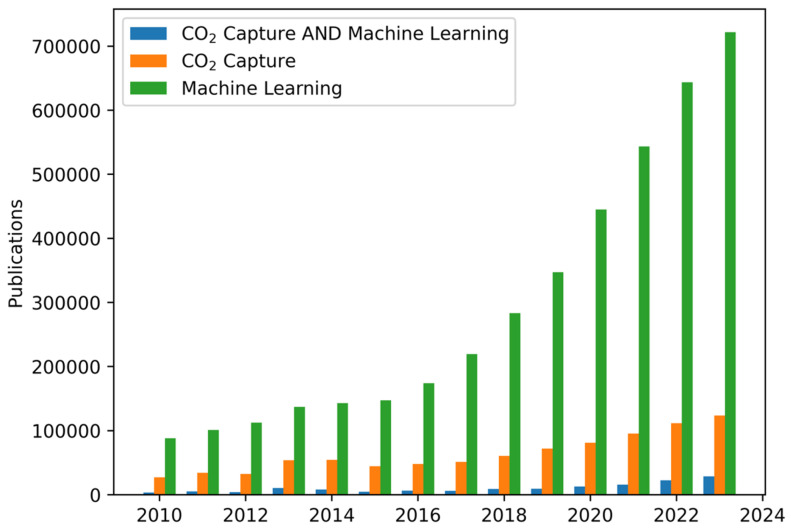
Number of publications on CO_2_ capture, ML, and the intersection of the two fields. Data gathered from Scopus, accessed 10 November 2024. Searches were limited to the fields of chemical engineering, chemistry, and material science. The ML query was conducted using the search term “Machine Learning”; the CO_2_ capture query was conducted using combinations of the following: (1) “CO_2_”, “Carbon”, or “Carbon Dioxide”; and (2) “Capture”, “Storage”, or “Sequestration”.

Computational methods such as molecular dynamics (MD) and Monte Carlo (MC) simulations have been cornerstones of the materials science field for decades; a noteworthy addition to these methods in this field is ML. While generative models [10] have garnered significant attention in recent years, language-based models [11] have also seen rapid development and a surge of interest. Artificial intelligence (AI) and ML are not limited to the applications that have captured the public’s imagination. The applications of ML and AI have been studied in a wide range of contexts ranging from art [12] and music [13,14] to finance [15,16,17], healthcare [18], and science [19,20]; their vulnerabilities [21] are yet another field of research. The number of research articles written on ML, CO_2_ capture, and their intersection has steadily increased (Figure 1). This paper reviews the effects ML has had on the discovery of materials for CO_2_ capture with a particular focus on the feature (also referred to as “descriptor”) categories used to describe candidate materials.

Considered a subfield of AI, ML is a computational method that utilises data analysis to predict outcomes and plays an important role in examining “big data” [22,23]. Since its first appearance, ML has been rapidly evolving and broadening the fields to which it is applied, including material science, in which it is becoming widely used for the identification and proposal of novel materials [24,25]. ML, fundamentally, aims to use a standard set of steps and analytical methods (i.e., an algorithm) to predict the output value for any given input. Depending on how the data are presented and what procedures are used, ML algorithms can be categorised into four categories: supervised, unsupervised, semi-supervised, and reinforcement [26,27,28]. Within these groups of algorithms, the ML models can be further categorised into classification (for predicting categorical variables) and regression (for predicting continuous variables), clustering, and data dimensionality reduction.

**Figure 2:**
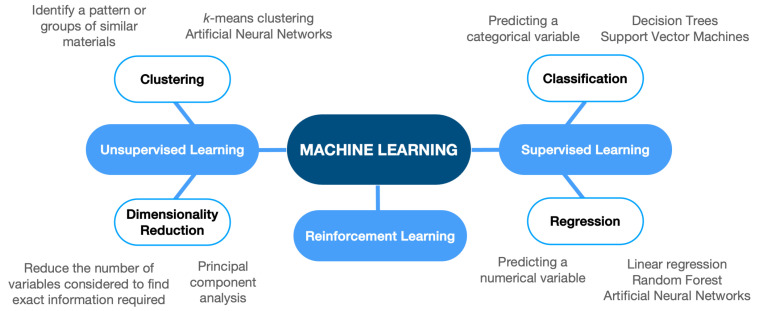
ML algorithms overview. ML algorithms grouped by overarching categories are shown in light blue ovals and more specific subcategories in white ovals. Examples of each subcategory’s algorithms and their function are provided in gray text above or below the relevant oval.

In their investigation into the state of the art of ML for carbon capture, utilisation, and storage (CCUS), Yan et al. [29] formed the categories shown in Figure 2. Among some of the most commonly utilised ML algorithms are linear regressions, logistic regressions, decision trees, naive Bayes, support vector machines, random forests, artificial neural networks, *k*-means clustering, hierarchical clustering, Gaussian mixture model, AdaBoost, and principal component analysis. Most of these algorithms, falling under the umbrellas of classification, regression, or both, can be used in various contexts, including ways to predict CO_2_-related material properties.

Yan et al. conclude that the distinct advantages of applying ML in CCS are that it provides the potential to identify links between data that are not readily identifiable, and it also provides alternative lower-computing-cost pathways. ML can be used in CCS to accelerate the design and development of materials for CCS purposes. The development of ML in CCS is expected to play a vital role in the acceleration of developing cost-effective CCS systems to tackle climate change. Being a data-driven method, it highlights the necessity for large quantities of data to develop generalised and robust ML models.

**Figure 3:**
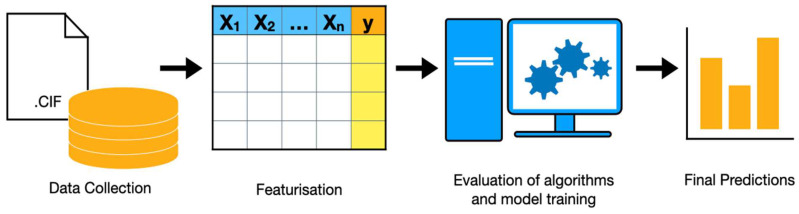
Illustration of the ML model building process. The process begins with the collection of data and structures, followed by gathering features for each material. The models are then trained, evaluated, and refined. Predictions are then made on unseen materials.

A simplified version of Yang et al.’s [30] illustration of the typical roadmap for developing and implementing an ML model is shown in Figure 3. A critical aspect of the ML process, included in the roadmap figure, is the development of descriptors. In the context of CO_2_ capture, Yang et al. describe featurisation as the process in which essential properties related to CO_2_ capture are transferred into numerical values that are readable by the computer. They proposed two broad subsets for these descriptors: external properties (e.g., the operating conditions and feed CO_2_ concentration) and intrinsic properties (e.g., physical and chemical characteristics). Being a crucial step in developing ML models for CO_2_ capture, this paper highlights the effectiveness of descriptor groups from both subsets.

ML for selecting CO_2_-capturing materials typically uses two approaches: (i) screening existing datasets and (ii) identifying trends and generating new structures not found in the existing datasets. While these two categories cover the main approaches to ML in CO_2_ capture, there are other aspects for which ML and computational methods come into play. Large language models are assisting researchers in their data collection and experimentation [31,32], genetic algorithms and other heuristic methods are being used to explore the hypothetical material search space, and MD and MC simulations are obviating the need for physically testing materials. This review mainly focuses on publications that have utilised ML models for evaluating materials in CCS applications.

## 2. Descriptors in ML for CO_2_ Capture

ML models can be built using experimental or simulated results; however, there is still a need to numerically or categorically describe the materials through descriptors so that an ML model can be built. Broadly, descriptors used to describe materials in CO_2_ capture datasets fall under the following categories: (a) operating conditions, (b) charge and orbital, (c) thermodynamic, (d) geometric and structural, and (f) chemical composition. Depending on the type of materials, application, and ML algorithm used, certain groups may be more prevalent in constructing the ML model. The prevalence can be determined through the algorithms’ internal ranking (if applicable) or through statistical methods such as Shapley values.

Considering carbon capture technologies, ML has slowly been utilised in both large-scale (industry) and small-scale (R&D and laboratory-scale) applications, including the deployment of solvent-based post-combustion capture [33], ionic liquids [34], adsorbents [35], and membranes [36]. In 2022, Hussin et al. conducted a bibliometric analysis and systematic review of ML approaches in carbon capture applications [28]. In the review, they provided a table of the most cited articles between 1999 and 2022; this was used as the starting point for evaluating the ML descriptors used in the most influential works in this review. Using the title, year, and ML model columns, the table was expanded to include a descriptors column to highlight the trends and scope of descriptors used when building ML models for CO_2_ capture (see Table 1).

The studies summarised in Table 1 vary significantly in terms of the domain to which ML is applied; while some are related to CO_2_ capture in materials, others look at larger, macro-scale settings and utilise considerably different descriptors than those used in the nano-scale settings of porous materials. MOFs, being assembled through distinct repeating units that can be algorithmically generated, are suitable candidates for a large range of descriptors. Clear boundaries of the repeating unit cell, known topologies, and secondary building units (SBUs) are grounds for gathering descriptors that would be inaccessible to materials with irregular, non-repeating patterns. MOFs have been studied extensively and have been used in many gas capture and storage contexts, including CO_2_ capture [47].

While MOFs are dominant in the field of CO_2_ capture materials, other candidates, such as ionic liquids and activated carbons, have been considered. Excluding reviews, publications with more than 10 citations (as of May 2024) that focus on the intersection of ML, CO_2_ capture, and material science are listed in Table 2. While polymers, silica, alumina, and other such materials have been used for CO_2_ capture, the use of ML for these materials is limited in this domain and are thus not listed in the table.

Beyond the articles listed above, other publications have utilised ML through generative models built using SMILES-based descriptors [53] and evaluated them through a reward function to design new CO_2_ capture materials or through ML-based analysis using more conventional descriptors, such as pore geometry- and chemistry-based features [54], to evaluate the diversity of structures in the dataset. While the specific descriptors may vary between publications, the overall information being captured through them can be grouped under broader categories. Most studies do not exclusively use a single group of descriptors; here, the descriptors used in the most-cited articles and other prevalent literature are discussed. The categories described aim to provide readers with an overview of this field in which, to the best of the authors’ knowledge, a descriptor-focused review of ML for CO_2_ capture has not been published.

### 2.1. Operating Conditions

Relevant at macro- (plant-based) and nano- (molecular-based) scales, operating conditions are key descriptors external to the adsorbent medium that determine the level of gas adsorbed. Daryayehsalameh et al., investigating 1-Butyl-3-methylimidazolium tetrafluoroborate [Bmim][BF_4_] ionic liquid, used only temperature and pressure as descriptors to build an ML model. The ionic liquid and its intrinsic properties remaining constant meant temperature and pressure were the only variables in the dataset [46]. The R^2^ displayed good agreement between the predicted values through ML and the reference values, thus proving the effectiveness of ML in predicting the CO_2_ capture of the material at varying external conditions. However, the similarity of conditions under which predictions were made could have played a considerable influence on the final result and would have inflated the predictive performance of the model.

By introducing more materials and a few material-specific descriptors, Baghban et al. [39] and Song et al. [44] succeeded at predicting CO_2_ capacity and solubility, respectively, in various ionic liquids and aqueous solutions. Rather than having separate temperature-specific or pressure-specific ML models, a single ML model that can make predictions on all operating conditions is beneficial. The feasibility of building such a model depends both on the algorithm’s robustness and the data used to fit the model. However, these studies by Baghban et al. and Song et al., by adding descriptors relating to the materials’ unique characteristics, were able to not only make predictions on an array of materials, but they were able to do so at varying operating conditions.

Leperi et al. [55], rather than searching for new materials, instead, focused on finding the appropriate pressure-swing adsorption (PSA) conditions for a given adsorbent material, which they describe as a challenge that must be addressed to make PSA commercially competitive for carbon capture applications. Focusing on Ni-MOF-74 and zeolite 13X, the team built an artificial neural network (ANN) model that utilises variables such as pressure, column temperature, and N_2_ loading as well as operating parameters such as molar feed rate, and column length to predict the column profile at the end of the feed step. They concluded that ANNs could be used as surrogate models for the rapid simulation of the individual steps of various PSA cycles. Looking at a similar macro-scale plant that utilises both adsorber and desorber columns, Sipöcz et al. [37] simulated a CO_2_ removal system based on a conventional amine absorption process. From the dataset compiled through the simulations, inlet flue gas temperature, inlet flue gas mass flow, inlet flue gas CO_2_ mass fraction, solvent lean load, solvent circulation rate, and removal efficiency were used as parameters to build an ANN that predicts the amount of CO_2_ captured, among other outputs. Using this model, the predictions of the CO_2_ captured had a maximum error that did not exceed 0.17%.

As evidenced by the studies discussed, when the adsorbent material or the macro-scale adsorption system remains constant, the role of pressure and temperature becomes critical. Unlike in ML models that search through libraries of candidate materials, in ML models built for a single material, pressure and temperature often become the only independent variables. As such, these models can be expected to have a much better performance than those looking at multiple materials. Often a simple interpolation or linear regression could yield performances sufficient for a desired application. The use of more advanced ML methods must, therefore, be scrutinized for such scenarios.

### 2.2. Charge- and Orbital-Based Descriptors

When more than one CO_2_-capturing material candidate is considered, it is crucial to describe the intrinsic characteristics of materials to allow the ML algorithms to distinguish between candidates. Adding material-specific characteristics allows researchers, through the use of statistical methods and ML, to find trends among the characteristics that make them suitable for CCS applications. Examples of such descriptors that are particularly important at lower partial pressures are charge-based and orbital-based descriptors. One example comes from Venkatraman and Alsberg, who considered applying ML to predict CO_2_ capture in ionic liquids (IL) [34]. A significant portion of their gas solubility data came from a review by Lei et al. [56], which summarised recent research produced for a number of ILs with different combinations of cations and anions. The highly tuneable nature of ILs was a leading factor in a large array of data being readily available. Data from other articles combined with the data from the review by Lei et al. resulted in the duo’s dataset containing over 10,000 IL- CO_2_ solubility datapoints. The study used molecular descriptors that were computed using the software KRAKENX [57]. Various quantum chemical and molecular orbital-based descriptors were calculated at the PM6 level of theory. Utilising a partial least squares regression (PLSR), conditional inference trees (CTREEs), and random forests (RFs), they trained algorithms using approximately 100 descriptors. Performing two 50:50 random splits on the dataset, they aimed to accurately assess the performance of the ML algorithms. The PLSR model performed considerably worse than the other two algorithms, with the PLSR model having an R^2^ of less than 0.5, while the RF and CTREE models had R^2^ values above 0.8.

While the CTREE algorithm highlighted the *charge partial surface areas* (CPSAs) as the most important descriptors, one of the molecular orbital descriptors was highlighted as the most important by the RF model. The authors highlighted that, in agreement with previous studies, CO_2_ adsorption is heavily influenced by the electron-donating ability, position, type, and number of substituents on the solvent. This aligns with the variables shown to be highly important as they correspond to geometrical and physicochemical properties of both cations and anions, in particular, those relevant to intermolecular interactions.

Furthermore, it was found that, according to frontier molecular orbital theory [58], the strength of the donor–acceptor interactions is typically determined by the overlap between the highest occupied molecular orbital (HOMO) on the donor and the lowest unoccupied molecular orbital (LUMO) on the acceptor. One such orbital-based descriptor was highlighted by the RF algorithm. The HOMO and LUMO energy descriptors are indicative of the cation–anion electrostatic interactions that are key to CO_2_ solvation abilities [59,60]. As a more comprehensive analysis on the topic of ILs (spanning 185 ILs), this study enforces the robustness of ML used to predict CO_2_ capture in another family of materials. The RF achieving an R^2^ up to 0.96 on the test set was found to be more accurate than those obtained with the quantum-chemistry-based COSMOtherm predictions.

Charge-based and orbital-based descriptors are not uncommon for ML models built for materials such as MOFs, in which charges can be encoded to the crystallographic files that represent the structures. Along with topological and chemical descriptors, Anderson et al. [42] included the highest dipole moment of functional groups in the MOF, the most positive charge, and the most negative charge in building ML models to predict CO_2_ adsorption. Other publications have created further descriptors to quantify the effects of each charge in an MOF framework through a polynomial function fitted to MC simulations using a fictitious massless atom of varying charges at different pressures [61]. Such descriptors, while improving ML models, require the structures to have the partial charges on atom sites readily available. As such, a drawback to using charge-based descriptors is the necessity of calculating the partial charges within the frameworks. While methods such as QEq are expedient, they lack the necessary precision. On the other hand, ab initio simulations based on density functional theory, while resulting in more accurate charge assignment on the framework, are more computationally expensive. Researchers, therefore, must weigh the costs and benefits of the trade-off between charge calculation methods before deriving any charge-based descriptors.

### 2.3. Thermodynamic Descriptors

Quantifying various aspects of adsorbent–adsorbate interactions, thermodynamic descriptors provide useful insight into CO_2_-capturing materials. Burns et al. [43], utilising the CoRE MOF dataset [62], conducted atomistic simulations fully integrated with a detailed vacuum swing adsorption simulator and validated them at the pilot-scale to screen 1632 experimentally characterised MOFs. A total of 482 materials were found to meet the 95% CO_2_ purity and 90% CO_2_ recovery targets (95/90-PRTs), 365 of which had parasitic energies with a low value of 217 kWh_e_/MT CO_2_ below that of solvent-based capture (∼290 kWh_e_/MT CO_2_). For post-combustion CO_2_ capture, they concluded that N_2_ rather than CO_2_ adsorption is found to be the key metric to predict whether a material can meet the 95/90 purity–recovery requirement. Using the adsorption metrics, ML models were built to predict whether a material can meet the 95/90-PRT. These models achieved an overall prediction accuracy of up to 91%. The CO_2_ and N_2_ uptake capacities, working capacities, heats of adsorption, and selectivities of each gas were among the descriptors used to build the models. Other studies have also shown thermodynamic descriptors, such as the Henry coefficient of CO_2_, to be important descriptors in ML predictions [61].

Thermodynamic descriptors are evidently highly influential; however, they can require significant computational resources. Therefore, similar to charge-based descriptors, weighing costs and benefits is required when considering the incorporation of thermodynamic descriptors. The appeal of ML methods is their ability to rapidly sift through large datasets and to lower the computational expense of evaluating materials. Some descriptors can only be derived through computationally expensive methods; in these cases, researchers must determine if calculating the metrics will be more beneficial than running simulations that calculate the target variable directly.

### 2.4. Geometric and Other Structural Descriptors

**Figure 4:**
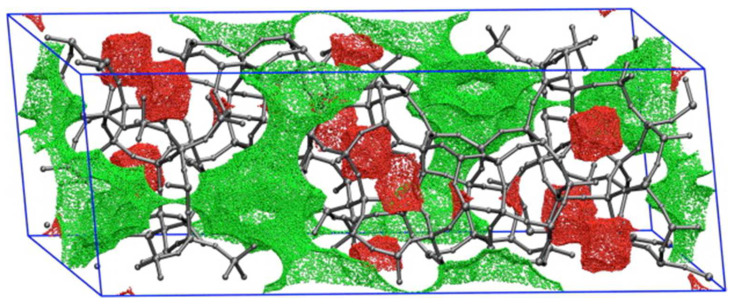
Sampled points on the surface of the DDR zeolite structure. Green and red points are accessible and inaccessible to a spherical probe of radius 3.2 Å, respectively [64].

As defined by the researchers who developed ToposPro (software used for topological analysis of crystal structures), the term *topology*, when applied to a chemical structure, carries the meaning of a set of chemical bonds or other links between structural groups (atoms, molecules, and residues) [63]. Therefore, any parameter that relates to these properties and characterises them can be considered topological. Geometric descriptors conventionally relate to, among other characteristics, the surface areas, volumes, channels within the structures, and pore characteristics (Figure 4). Geometric descriptors such as these can be considered a consequence of the composition of the materials’ building units (including the presence of functional groups located at nodes or linkers) and the topology; with that being the case, topological, structural, and geometric descriptors were grouped under the same header in this paper.

**Figure 5:**
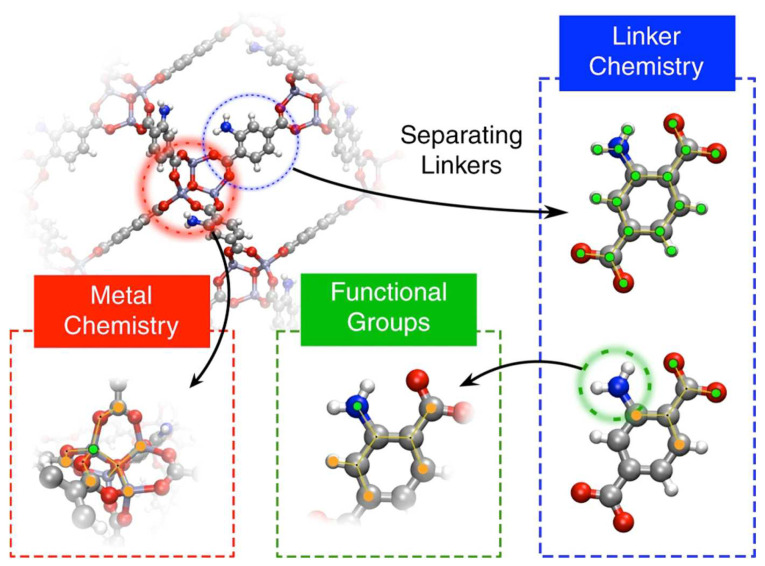
Description of the three domains of MOF chemistry by Moosavi et al. [74]: Metal centre revised autocorrelations (RACs) are computed on the crystal graph. Linker and functional-group RACs are computed on the corresponding linker molecular graph. Linker chemistry includes two types of RACs, namely full linker and linker connecting atoms. The graphs show the start atom (in green) and the nearby atom (in orange) used to define the RAC descriptors.

MOFs’ discrete repeating structures provide grounds for a wide array of topological and structural descriptors to be derived. Focusing on the MOF family of materials, Anderson et al. [42] computationally synthesised over 400 MOFs using ToBaCCo [65] by combining MOF SBUs. Calculating the adsorption loading in MOFs via grand canonical Monte Carlo (GCMC) simulations using the RASPA code [66], gathering geometrically calculated descriptors through Zeo++ [64] (accessible and inaccessible surface areas, as exemplified in Figure 5), and other descriptors such as the highest dipole bond moment led to the team compiling a dataset containing over 3000 simulation results.

Utilising the R programming language, the team implemented the following ML algorithms: multiple linear regression (MLR), support vector machine (SVM), conditional inference decision trees (DTs), random forests (RFs), neural networks (NNs), and gradient boosting machines (GBMs). Functionalisation affecting CO_2_ adsorption was highlighted, focusing on the pores specifically. The authors reported that some functionalisations result in a pore chemistry that typically enhances CO_2_ loadings relative to the parent MOF, whereas some other functionalisations usually have the opposite effect. Further insight into the topological effects was provided through the use of ML.

GBM models were then used to investigate optimal chemical and topological property combinations for CO_2_ capture metrics, and a genetic algorithm was used to maximise the predictions of all five metrics for each topology by the GBM models. The study found that targeting an LPD of ~nine Å and a PLD of ~four Å would yield desirable performances in the metrics the team considered. It was also found that to maximise working capacities, much higher VSA values were needed than to maximise selectivities and pure CO_2_ loading. Additionally, the team noted that the selectivities and working capacities of CO_2_:N_2_ required higher bond dipole moments of the functional groups than for the CO_2_:H_2_ mixture. The use of genetic algorithms in combination with other ML algorithms, as demonstrated by the study, is an example of how design strategies can be derived through such computational methods.

Ma et al. applied ML to study the effects of pore structure, chemical properties, and adsorption conditions on CO_2_ adsorption performance based on 1594 CO_2_ adsorption data points gathered from the previously published literature [67]. A distinguishing feature of this paper is that the group did not focus on a single, niche family of materials but considered a broader category, termed porous carbons. These included MOFs, porous organic polymers, biomass (e.g., tobacco stem and glucose), and organic salt, among others. In their study, they selected random forest as their ML algorithm due to its proven track record in predicting heavy metal adsorption by biochar [68] and the adsorption of CO_2_/CH_4_ by various types of coal [36]. Using R^2^ and RMSE as their performance metrics, the team utilised a *k*-fold cross-validation technique (70% train and 30% test) to tune the parameters of the ML model. Because the dataset consisted of adsorption data at varying temperatures and pressures, the adsorption conditions were added as descriptors alongside pore structure and chemical properties.

They found that, depending on the adsorption conditions of the test set, the model’s performance ranged from an R^2^ of 0.978 to 0.995, with RMSEs ranging from 0.057 to 0.150 mmol/g. These ML results are important for the topic of CO_2_ capture in two important ways: (i) the study demonstrates how the existing literature on a collection of different materials can be effectively utilised in an ML model; and (ii) the paper provides actionable insight into trends that enable high CO_2_ uptake through a feature importance analysis of the ML algorithm, experimental verification, and molecular simulations. At pressures below 0.5 bar, the study concluded that pressure was an important factor in predicting the adsorption of CO_2_, while its influence waned as the pressure increased. It was also found that CO_2_ adsorption density decreased with increasing pore size; this was attributed to smaller pores providing a stronger adsorption potential between the pore wall and the gas molecules [69]. Finally, they concluded that the prevalence of functional groups also plays a role in a material’s adsorption capacity; the doping of oxygen and nitrogen groups can enhance CO_2_ adsorption capacity.

### 2.5. Chemical Composition-Based Descriptors

The elements and chemical features that compose a structure are characteristics that vary widely between materials and are thus ideal for creating descriptors for ML algorithms to discern between materials. The creation of chemical descriptors has varied in both approach and context [70,71]. Pardakhti et al., while predicting methane adsorption in MOFs, created chemical descriptors that relied on the number and ratio of elements present in the adsorbent. This descriptor was later used in other contexts, including CO_2_ capture [61,72,73]. Moosavi et al., on the other hand, wanted to inspect the structural building blocks of MOFs rather than an expedient but less informative approach. They analysed the contents of the complete structure (Figure 5). Simulating the CO_2_ adsorptions at 0.15 bar and 16 bar for CO_2_ and utilising three databases of MOF structures, the team trained ML models based on the chemistry of the metal nodes, the chemistry of the linkers, and the functional groups as the chemical descriptors. Along with the chemical descriptors, the descriptors relating to the pore geometry were used in the training of the ML models. The study obtained a Spearman rank correlation coefficient (SRCC) of above 0.9. It was noted that for properties that are less dependent on the chemistry (e.g., high-pressure applications), the geometric descriptors are sufficient to describe the materials with the average relative error. However, for applications in which chemistry plays a role, such as predicting the Henry coefficient of CO_2_, the chemical descriptors are essential to accurately predict the material properties. Similarly, predicting the maximum positive charge and minimum negative charge was possible using the chemical descriptors but not the geometric descriptors.

Using a similar algorithm to that introduced by Wilmer et al. [75], the authors generated approximately 1800 unfunctionalised base structures, combining 66 SBUs and 19 functional groups, resulting in a total of 324,500 hypothetical MOF structures. Utilising SVMs, the structures in the test set were predicted as having either a high or low uptake (by determining a cutoff value). Depending on the threshold used, the algorithm could discard between 67% and 95% of the dataset, greatly reducing the number of candidate materials to be simulated. The study derived the AP-RDF descriptors that would encapsulate the chemistry of periodic structures such as MOFs. It was suggested that the AP-RDF profile of an MOF framework could be interpreted as the weighted probability distribution to find an atom pair in a spherical volume of radius R inside the unit cell. In their work, the use of classifiers necessitated the employment of receiver operating characteristic (ROC) plots to evaluate their performance. The area under the curve (AUC) of the ROC plot provides a basis for comparing classifiers. The AUC values range between 0 and 1, where AUC = 1 indicates a perfect classifier. The AUCs of the ML models trained in this study were 0.979 and 0.978 for the 0.15 and 1 bar classifiers, respectively, when predicting CO_2_ capture. Dureckova et al. [48] used this descriptor in their work to predict both the CO_2_ capacities (R^2^ = 0.944) and CO_2_/H_2_ selectivities (R^2^ = 0.872).

Moosavi et al. utilised regression models to predict the heat capacity of materials for CO_2_ capture [76]. The models were built using atomic, geometric, and chemical descriptors, and it was able to yield good agreement between the predictions and DFT simulation results (Table 2). Their simulations on ZIFs showed that changes in the topology have a minor effect on the heat capacity; thus, it was concluded that the relevant chemical environment is relatively short-ranged; this led the team to consider only the local environment surrounding the atoms in a framework, rather than the complete structure for chemical features. Achieving a good performance from a model based on these descriptors, the study showed that it was not only possible to predict the heat capacity of materials but also that the chemical featurisation of these materials could be achieved by considering only the local environment.

## 3. Descriptor Selection Strategies

As highlighted in the previous section, numerous descriptors can be used to represent the candidate materials; however, only a limited number are significantly relevant to the target property [77]. Feature selection algorithms enhance model performance by reducing the dimensionality of the descriptor space to prevent overfitting and decrease training time by minimising the number of input variables [70]. Feature selection algorithms can be categorised into the filter, wrapper, and embedded methods [71].

The filter method intuitively ranks features based on their relevance to the property and selects the top ones. A feature’s relevance can be quantified using metrics such as the Pearson correlation coefficient, Spearman’s rank correlation coefficient, and Kendall’s tau. In the study by Venkatraman and Alsberg [34], using only descriptors highly correlated with the target variable, the authors built an ML model that screened suitable ILs.

Instead of selecting descriptors individually based on their relevance, the wrapper method aims to identify a subset of descriptors that collectively optimise the performance of the ML models [78]. This method is ideal for achieving a high accuracy with a specific model [79] while having the drawback of substantial computational costs. In their study [80], Dong-Hoon Oh et al. used a stepwise backward elimination algorithm to select the best descriptor set to successfully predict the CO_2_ capture potential of amine-based capture processes.

The embedded method is a combination of filter and wrapper methods. Once a preliminary set of relevant features is established, these features are then used in conjunction with ML models. During the training process, the model evaluates the performance of different subsets of selected features. By incorporating regularisation techniques, such as Lasso or Ridge regression, the embedded method can further refine the descriptor set by penalising less important features, thereby promoting the selection of a more optimal subset. Through iterative training and validation, the embedded method identifies the best-performing feature subset that maximises model accuracy while minimising complexity. This method can reduce the computational cost and enhance the algorithm efficiency, becoming the main choice for feature selection algorithms [81].

## 4. Machine Learning Model Optimisation

### 4.1. Hyperparameter Tuning

The optimisation of hyperparameters in ML models is a crucial step in enhancing model performance [82]. Hyperparameters are values that configure the learning process, and selecting an appropriate set is essential for achieving optimal results. An appropriate optimisation algorithm can significantly enhance the model’s performance [77].

Manual testing is the most direct strategy among the hyperparameter tuning methods, in which researchers select and test hyperparameters based on intuition and experience. The effectiveness of hyperparameters often depends on how well they align with the chosen descriptors, as these descriptors shape the input features that the model relies on for learning. While this fast approach allows researchers to gain insights into how a model is affected by different hyperparameters, the method is unreliable in finding the best hyperparameters for a given ML model, and it relies heavily on an understanding of ML models. The relationship between hyperparameters and model performance is complex; it can therefore be time-consuming for researchers to find the hyperparameters that lead to a sufficient performance for the desired application. In the study by Xiaomei Deng et al. [83], hyperparameters such as the training function and transfer were set manually. The study, utilising the RF algorithm, successfully identified 14 MOFs with optimal CO_2_ adsorption and diffusion properties. In the study by Yue Jian et al. [84], the authors manually set the hyperparameters for their graph convolutional network model, including the dimension of four convolutions, the activation algorithm, and the dropout ratio. Their model, utilising the graph neural network (GNN), achieved a mean absolute error (MAE) of 0.0137 and an R^2^ value of 0.9884.

In contrast to manual testing, the grid search algorithm exhaustively explores a specific range of values for hyperparameters [85]. All possible hyperparameter combinations within the given range of values form a grid that can, in turn, be tested to determine the best hyperparameter combination. The model is trained and evaluated based on each combination in the grid and the best performing one is selected. A grid search can find the best hyperparameter combination within the tested combinations but requires significant time and computational resources to do so. This is, therefore, more suitable for smaller hyperparameter sets. In the publication by Jake Burner et al. [86], a grid search was conducted to optimise the dropout probability, the number of hidden layers, the number of nodes in each hidden layer, and the learning rate. The optimized model predicted for CO_2_ working capacity and CO_2_/N2 selectivity in MOFs under low-pressure conditions. It achieved R^2^ values of 0.96 for CO_2_ working capacity and 0.95 for selectivity, successfully identifying 994 of the top 1000 MOFs from a test set, thereby demonstrating over a tenfold speed-up for pre-screening materials for more computationally intensive simulations.

In contrast to the systematic approach of the grid search method, a random search is a technique that avoids the computational expense of the grid search method by randomly sampling the hyperparameter search space [87]. While it can reduce computational expenses, the randomness of the algorithm cannot guarantee the best combination will be found.

### 4.2. Evaluation of the Performance of Models

Evaluation metrics are measurements used to quantify the performance of ML models [88]. They offer insights into how closely a model’s predictions match actual outcomes, allowing the assessment of the model’s effectiveness, accuracy, and reliability. These metrics help guide decisions related to model selection, tuning, and optimization [89].

#### 4.2.1. Coefficient of Determination

R square (R^2^) is a statistical measure used to determine how well the regression model prediction fits the data [90]. R^2^ values range from zero to one, and a higher value indicates a better fit of the predictions. R^2^ represents the relative relation between the original data and a prediction and, thus, is more informative than other metrics counting absolute errors, such as MAE and RMSE [91].

(1) $$R^{2}=1-\frac{\sum_{i=0}^{n}(y_{i}-\hat{y_{i}}{)}^{2}}{\sum_{i=0}^{n}(y_{i}-\bar{y}{)}^{2}}$$

Equation (1) demonstrates the method for calculating R^2^ for predictions made using a regression model. In the equation, *n* denotes the count of data, *y_i_* denotes the original data, $\hat{y_{i}}$ denotes the predictions, and $\bar{y}$ denotes the average value of the original data.

#### 4.2.2. Mean Absolute Error

The mean absolute error (MAE) calculates the average gap between the original data and predictions. As this is a measure of error, more precise predictions are closer to zero [92]. Equation (2) demonstrates the method for calculating MAE.

(2) $$MAE=\frac{\sum_{i=1}^{n}|y_{i}-\hat{y_{i}}|}{n}$$

where *n* denotes the data count, *y_i_* denotes the original data, and $\hat{y_{i}}$ denotes the predictions.

#### 4.2.3. Root Mean Square Error

The root mean square error (RMSE) measures the square root of the average squared differences between predicted values and original values, measuring how well the model’s predictions align with the observed data [93]. Similar to MAE, the closer to zero, the more accurate the prediction. However, the squaring of the difference between the true and predicted values results in outliers having a greater impact on the metric.

(3) $$RSME=\sqrt{\overset{n}{\underset{i=1}{\sum }}\frac{(y_{i}-\hat{y_{i}}{)}^{2}}{n}}$$

#### 4.2.4. Recall Rate

The recall rate is a metric used in classification tasks to measure the ML model’s ability to correctly identify instances [94]. It focuses on how well the model identifies positive cases and is particularly important when missing positive instances have significant consequences. The recall rate ranges from zero to one, with values closer to one indicating a better performance in identifying positive instances. It is calculated as follows:

(4) $$Recall=\frac{TP}{TP+FN}$$

Here *TP* (true positives) is the number of correctly predicted positive instances and *FN* (false negatives) is the number of actual positive instances that the model incorrectly classified as negative.

#### 4.2.5. Spearman’s Rank Correlation Coefficient

Spearman’s rank correlation coefficient (SRCC) is a non-parametric measure of rank correlation that assesses the strength and direction of the monotonic relationship between two ranked variables. Its values range from negative one to one. A high positive SRCC (close to one) indicates a strong positive correlation, a value near zero suggests no correlation, and a value close to negative one represents a strong negative correlation [95]. It is calculated as shown in Equation (5).

(5) $$\rho =1-\frac{6\sum d_{i}^{2}}{n(n^{2}-1)}$$

where *d* is the difference between the ranks of the corresponding values of the two variables.

#### 4.2.6. Average Absolute Relative Deviation

The average absolute relative deviation (AARD) is a metric that measures the average relative difference between predicted and actual values, indicating how distant the predicted values are to the actual values [96].

(6) $$AARD=\frac{1}{n}\overset{n}{\underset{i-1}{\sum }}|\frac{y_{i}-\hat{y_{i}}}{y}|$$

*AARD* can be calculated through Equation (6). Lower AARD values indicate a better performance by the model; AARD values can be as low as zero; however, there is no upper limit to this value.

Depending on the application, researchers may opt to use one metric over another. As highlighted by Alexander et al. [90], even the commonly used metric of R^2^ has its shortcomings; they encourage the reporting of root mean square error or equivalent measures of dispersion, which they argue is of more practical importance than R^2^. Opting for one evaluation metric over another or removing certain datapoints from a dataset without true justification has the possibility of falsely boosting the model’s performance.

### 4.3. Descriptor Importance and Design Strategies

The reticular nature of MOFs and Zeolites, along with their virtually infinite number of possible configurations, allows for the production of descriptors that would otherwise be difficult. Distinct and identifiable topologies, SBUs, and tuneable functional groups within frameworks all contribute to these materials having expansive lists of descriptors. As with most adsorbents, the adsorption metrics such as permeability and heat of adsorption could be used for these materials as well; however, these descriptors are typically more expensive (computationally or materially) to obtain. MOFs, in particular, have been studied extensively for CO_2_ capture purposes, and based on the descriptors used, certain design strategies have been derived, such as including specific functional groups, targeting specific pore sizes, and utilising pre-determined topologies.

Ionic liquids, on the other hand, have fewer readily available descriptor types. Daryayehsalameh et al., when investigating the solubility of CO_2_ in 1-n-butyl-3-methylimidazolium tetrafluoroborate [46], utilised only the operating conditions (temperature and pressure) as the descriptors. Since the material remained constant, these were the only independent variables and thus were the only ones necessary to build the model. Due to similar conditions, the model’s performance was inflated compared to those that made predictions on a wide range of candidate ILs. ML models considering more than a single ionic liquid could include thermodynamic, geometric, structural, and chemical descriptors to differentiate between the ILs. Categorical descriptors for the anions and cations, SMILES-based descriptors, and molecular fingerprints could also be included in the featurisation of these materials. For direct CO_2_ capture, intermolecular cation–anion interactions were found to be important [34], while for CO_2_/N_2_ selectivity and permeability, accessible volume and accessible surface area were shown to have the greatest relative importance in the building of ML models [35]. Publications on the use of ML to predict CO_2_ capture properties in other materials remain limited; however, the available literature on such materials indicates the use of similar descriptors.

**Figure 6:**
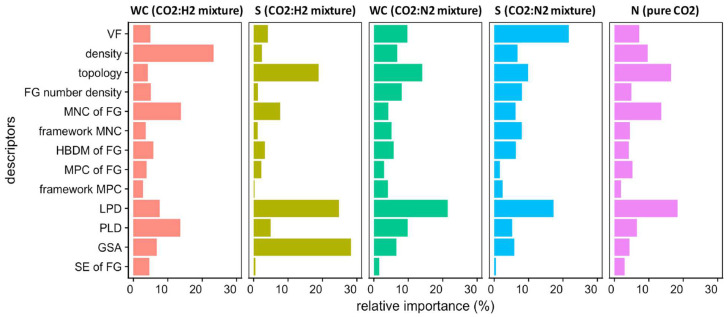
Influence of descriptors for predictions in various contexts [42], where the feature group *S* denotes selectivity, WC denotes working capacity, N denotes adsorption loading, FG denotes functional group, VF denotes void fraction, HDBM denotes highest dipole moment, MPC denotes most positive charge, MNC denotes most negative charge, LPD denotes largest pore diameter, PLD denotes limiting pore diameter, SE denotes sum of epsilons, and GSA denotes gravimetric surface area.

It is not just the materials in the dataset that influence feature importance; the context in which models are built also plays a role. Anderson et al. [42] demonstrated the varying relative importance of descriptors based on the type of ML models (Figure 6). In some instances, a specific descriptor can be an overwhelming contributor to the predictions made. For example, in some studies, it was shown that the importance of the heat of adsorption for predicting selectivity in a model utilising only six descriptors was significantly greater than all others.

**Figure 7:**
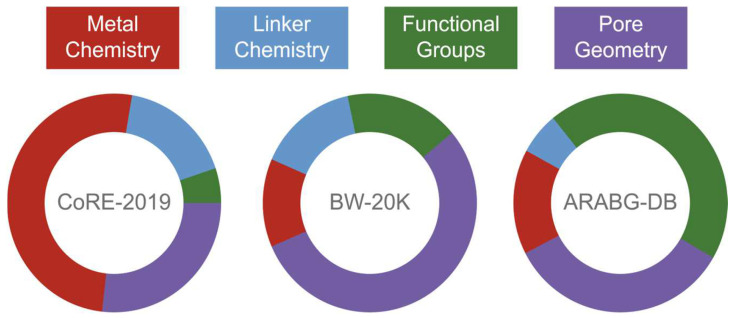
The relative importance of feature groups for CO_2_ adsorption predictions adapted from Moosavi et al. [74], based on the work by Rahimi et al.[97]. The charts depict the relative importance of the metal chemistry (shown in red), linker chemistry (shown in blue), functional groups (shown in green), and pore geometry (shown in purple) descriptor groups for making predictions on the CoRE MOF, BW-20 K, and ARABG databases [42,62,98].

Moosavi et al. [74], utilising three datasets, made predictions on CO_2_ uptake in MOFs. The ML algorithm, finding different trends in each dataset, gave different weights to the importance of the descriptor group (Figure 7). Taking into account how the values are distributed for the descriptor categories and their relative importance in the prediction-making process, inferences can be drawn to derive design strategies.

Trained using different datasets, studying different working conditions, or for different applications entirely, ML will display greater benefits from varying descriptors. In the context of CO_2_ capture, external conditions were shown to be influential for both macro-scale (e.g., CCUS plants) and nano-scale (e.g., MOF) systems. Acknowledging the existence of other descriptor categories, among the most used in ML for CO_2_ capture materials were the chemical, topological, thermodynamic, and charge/orbital-based descriptors.

An essential utility of trained ML models is to derive design strategies for specific applications; this can be guided by the knowledge of which descriptors are most important; however, it is not always necessary. A simple approach is to screen out optimal candidates through accurate predictions and to find common traits among the screened group. This approach is intuitive and easy to apply, but the diversity and volume of the candidate set will significantly influence the outcome. Researchers can manually select promising candidates, such as when Xiangyu Zhang et al. manually selected 10 combinations of metal nodes and topologies [53].

Genetic algorithms are another approach to deriving design strategies [99]. These algorithms generate a diverse population of candidate structures, each representing a potential solution to the optimisation problem. The candidate structures are evaluated based on a fitness function, which measures how well they meet the desired criteria. Through selection, crossover, and mutation, the population evolves over multiple generations, gradually improving the quality of the solutions. A design strategy can be derived by observing which traits are selected through the generations.

Another approach is to derive trends through an interpretable ML model. Model interpretation aims to provide transparency in decision making, revealing how input descriptors influence the outcomes of the models. This approach offers valuable insights into the underlying relationships between features and the target variable. In the context of CO_2_ capture, ML model interpretation can offer a clearer understanding of how various descriptors contribute to the CO_2_ capture capacity. The article by Jian Guan et al. [100], provides an example of such an application, in which their team determined that a pore size greater than one nm and a surface area of approximately 800 m^2^g^−1^ were necessary for their desired application.

The quality of data in the dataset used plays an important role, both in the accuracy of the model and in which descriptors are highlighted as being the most important in capturing CO_2_. Inaccurate models caused by low-quality data can lead to imprecise and flawed design strategies being derived. Therefore, to gain true insight through descriptors, diligence is required in scrutinizing not only the methods used (such as ML models and evaluation metrics) but also the data quality.

## 5. Conclusions and Perspectives

This paper reviewed the use of ML in CO_2_ capture with a specific focus on the descriptors used. ML is becoming an ever-increasingly important tool in evolving the CCS technology landscape. New approaches are frequently considered for creating descriptors relating to gas capture and storage [101].

MOFs, zeolites, and ionic liquids currently dominate the research into applications of ML on carbon-capturing materials. Some publications have demonstrated the effectiveness of using only thermodynamic conditions to make predictions on a specific material’s CO_2_-capture properties. In datasets involving more than one material, structural, chemical, adsorption metric-based, and charge-based descriptors have all played roles in predicting CO_2_ capture to varying levels of importance. Applicable also to MOFs, SMILES-based and molecular fingerprint descriptors were among those used in the most cited ML studies focusing on ionic liquids [102].

Beyond the ability to predict which materials would be most suitable for the application on which the ML model was trained, trained models can provide insight into which descriptors play a greater role in the suitability of materials; design strategies can be derived with such insights. Researchers must pay close attention to which metrics are used to evaluate the performance of ML models; overstating the performance of an ML model is possible by favouring certain metrics over others. Therefore, it is critical to examine whether ML models would truly meet the expectations for a given application.

We speculate that the breadth of materials evaluated through ML will continue to increase, and novel approaches to featurising materials will continue to be developed to effectively represent and differentiate data points within a dataset. Materials such as eutectic solvents [103] are examples of emerging materials in CO_2_ capture that can utilise ML. The overlap of material science, CO_2_ capture, and ML is an area of research that will undoubtedly continue to expand. All of these factors will allow for the intersection of CO_2_ capture technologies and ML to amplify. The domain of ML applications is rapidly enlarging, and as such, the role it plays in designing and evaluating CO_2_ capture materials will gain further importance; through generative AI methods, research into ML for the generation of materials is already being conducted [104].

What will undoubtedly guide the adoption of materials discovered through ML and other computational methods is their feasibility in synthesis. The cost of synthesizing discovered materials and the stability of those materials will determine whether scaling the production of any candidate highlighted by ML is feasible. Affecting the economic aspect of this field of research is whether useful byproducts can be generated through the captured CO_2_; formic acid is one such material that can be generated from the captured CO_2_ if it can be efficiently released and used in its formation.

## Figures and Tables

**Table 1:** Most cited articles on ML + CO_2_ capture between 1999 and 2022, compiled by Hussin et al. [28]. Publications are shown in descending order based on the number of citations at the time of compiling the table. The columns highlight the various machine learning algorithms and descriptors used [37,38,39,40,41,42,43,44,45,46].

Title	Year	Type of Machine Learning	Descriptors
The use of Artificial Neural Network models for CO_2_ capture plants	2011	ANN	Temperature, Mass Flow, Mass Fraction, Solvent Lean Load, Solvent Circulation Rate, Removal Efficiency
Rapid and Accurate Machine Learning Recognition of High Performing Metal Organic Frameworks for CO_2_ Capture	2014	Support Vector Machine (SVM)	Chemical Descriptors via Atomic Property-Weighted Radial Distribution Function (AP-RDF)
Rigorous modelling of CO_2_ equilibrium absorption in ionic liquids	2017	Least Square Support Vector Machine, Adaptive Neuro-Fuzzy Inference System, Multi-Layer Perceptron Artificial Neural Network, and Radial Basis Function Artificial Neural Network	Operating Temperature, Operating Pressure, Critical Temperature, Critical Pressure, Acentric Factor
Prediction of storage efficiency on CO_2_ sequestration in deep saline aquifers using artificial neural network	2017	ANN	Porosity, Thickness, Permeability, Depth, Time, Residual Gas Saturation
Prediction of CO_2_ loading capacities of aqueous solutions of absorbents using different computational schemes	2017	MLP-ANN, Radial Basis Function ANN, LSSVM, and ANFIS	Temperature, Concentration, Molecular Weight, Pressure
Role of Pore Chemistry and Topology in the CO_2_ Capture Capabilities of MOFs: From Molecular Simulation to Machine Learning	2018	Multiple Linear Regression, SVM, Decision Trees, Random Forests, Neural Networks, and Gradient Boosting Machines.	Functional Group (FG) Number Density, Void Fraction, Highest Dipole Moment of FG, Most Positive Charge, Most Negative Charge, Largest Pore Diameter, Limiting Pore Diameter, Sum of Epsilons, Gravimetric Surface Area
Prediction of CO_2_ solubility in ionic liquids using machine learning methods	2020	ANN and SVM	Temperature, Pressure, Building Groups (similar to SBUs)
Prediction of MOF Performance in Vacuum Swing Adsorption Systems for Post-combustion CO_2_ Capture Based on Integrated Molecular Simulations, Process Optimizations, and Machine Learning Models	2020	ANN and Gradient-Boosted Decision Tree Model	Adsorption Metrics (e.g., Henry’s Selectivity, Heat of Adsorption, Working Capacity), Geometric Descriptors
Machine learning exploration of the critical factors for CO_2_ adsorption capacity on porous carbon materials at different pressures	2020	Random Forest	Textural Properties, Chemical Composition, Pressure
Modeling of CO_2_ capture ability of [Bmim][BF4] ionic liquid using connectionist smart paradigms	2021	ANN, Cascade Feed-Forward Neural Network, SVM, ANFIS	Temperature, Pressure

**Table 2:** Most cited articles for machine learning in CO_2_ capture materials as of May 2024, separated by material category, providing the title of the article, ML algorithm used, descriptors used, the target variable being predicted, and the most relevant performance metrics [34,35,38,42,43,46,48,49,50,51,52].

Material	Title	Descriptors	Algorithm	Performance	Target
MOF and Zeolite	Rapid and Accurate Machine Learning Recognition of High Performing Metal Organic Frameworks for CO_2_ Capture	AP-RDF	SVM	Up to 99.9% recall rate of the top 1000 MOFs at 0.15 bar and 96.8% at 1 bar	Classification of MOF CO_2_ adsorption capacity (>1 mmol/g at 0.15 bar and >4 mmol/g at 1 bar)
	Role of Pore Chemistry and Topology in the CO_2_ Capture Capabilities of MOFs: From Molecular Simulation to Machine Learning	Topological, geometric, charge-based	DT, RF, MLR, GBM, SVM, NN	[CO_2_/N_2_ Selectivity] R^2^ = 0.905, SRCC = 0.921 [CO_2_ Loading] R^2^ = 0.905, SRCC = 0.950, [CO_2_/H_2_ selectivity] R^2^ = 0.855, SRCC = 0.938	CO_2_ loading, CO_2_/N_2_ selectivity, CO_2_/H_2_ selectivity
	Prediction of MOF Performance in Vacuum Swing Adsorption Systems for Postcombustion CO_2_ Capture Based on Integrated Molecular Simulations, Process Optimizations, and Machine Learning Models	Geometric, adsorption Metrics, figures of merit (Yang’s FOM, Wiersum’s FOM, etc.)	Random Forest	[Productivity] Correlation R^2^ = 0.41 [PE] Correlation R^2^ = 0.18	Productivity of a material (i.e., how much CO_2_ the sorbent can extract per unit volume of the material per unit time), parasitic energy
	Robust Machine Learning Models for Predicting High CO_2_ Working Capacity and CO_2_/H_2_ Selectivity of Gas Adsorption in Metal Organic Frameworks for Precombustion Carbon Capture	Geometric, AP-RDF	Gradient boosted trees	[CO_2_ working capacities] R^2^ = 0.944 [CO_2_/H_2_ selectivities] R^2^ = 0.872	CO_2_ working capacities, CO_2_/H_2_ selectivity
	A data-science approach to predict the heat capacity of nanoporous materials	Geometric, atomic, chemical	XGB	MAE = 0.02 RMAE = 2.89% SRCC = 0.98	Heat capacity (J g^−1^ K^−1^)
	Large-Scale Screening and Machine Learning to Predict the Computation-Ready, Experimental Metal-Organic Frameworks for CO_2_ Capture from Air	Five structural parameters: volumetric surface area (VSA), largest cavity diameter (LCD), pore-limiting diameter (PLD), porosity φ, density ρ, and an energy parameter: heat of adsorption	BPNN, RF, DT, SVM	Train R = 0.994, Test R = 0.981 (RF Model)	Adsorption selectivity (CO_2_/N_2_+O_2_)
	Design and prediction of metal organic framework-based mixed matrix membranes for CO_2_ capture via machine learning	Operating conditions, polymer type, geometric, gas adsorption metrics (selectivity, permeability, etc.)	RF	[Permeability] R^2^ = 0.77, RMSE = 1.45 [Selectivity] R^2^ = 0.7, RMSE = 0.31	CO_2_ permeability, CO_2_/CH_4_ selectivity
	High-Performing Deep Learning Regression Models for Predicting Low-Pressure CO_2_ Adsorption Properties of Metal Organic Frameworks	AP-RDF, chemical motif, and geometric descriptors	ANN (MLP)	[CO_2_ working capacity] Pearson r^2^ = 0.958, SRCC = 0.965, RMSE = 0.13 [CO_2_/N2 selectivity] r^2^ = 0.948, SRCC = 0.975, RMSE = 10	CO_2_/N_2_ selectivity
Ionic Liquids	Modeling of CO_2_ capture ability of [Bmim][BF_4_] ionic liquid using connectionist smart paradigms	Temperature, pressure	ANN, LS-SVM, ANFIS	AARD (%), MSE, RMSE, R^2^ = 7.01, 0.00115, 0.03396, 0.98408	Solubility of CO_2_ in the 1-n-butyl-3- methylimidazolium tetrafluoroborate ([Bmim][BF_4_])
	Predicting CO_2_ capture of ionic liquids using machine learning	Semi-empirical (PM6) electronic, thermodynamic, and geometrical descriptors	SVM, RF, XGB, MLP, graph-based networks	[Dataset-1] R^2^, RMSE (MAE) = 0.96 0.05 (0.03) [Dataset-2] R^2^, RMSE (MAE) = 0.85 0.10 (0.06)	CO_2_ solubility in 1-Butyl-3-methylimidazolium hexafluorophosphate ([Bmim][PF_6_])
	Prediction of thermo-physical properties of 1-Butyl-3-methylimidazolium hexafluorophosphate for CO_2_ capture using machine learning models	Temperature, CO_2_ partial pressure and water wt%	Gaussian process regression	R^2^ = 0.992; AARD% = 0.137976	CO_2_ solubility in [Bmim][PF_6_]
	Machine learning aided high-throughput prediction of ionic liquid@MOF composites for membrane-based CO_2_ capture	Structural and chemical	RF	R^2^ = 0.728, RMSE = 0.365, MAE = 0.277	CO_2_/N_2_ Selectivity
	Predicting CO_2_ Absorption in Ionic Liquids with Molecular Descriptors and Explainable Graph Neural Networks	Morgan fingerprints, temperature, pressure	PLSR, CTREE, RF	MAE of 0.0137, R^2^ of 0.9884	CO_2_ absorption/solubility in ILs
Others (Graphene, Graphite, and Activated Carbon)	Monitoring the effect of surface functionalization on the CO_2_ capture by graphene oxide/methyl diethanolamine nanofluids	Temperature, pressure, functionalized group, graphene oxide dosage	CFF-NN	AARD = 1.78%, MSE = 0.007, RMSE = 0.08, and R^2^ = 0.9906	CO_2_ solubility in graphene oxide/methyl diethanolamine
	Intelligent prediction models based on machine learning for CO_2_ capture performance by graphene oxide-based adsorbents	Geometric (surface area, pore volume), temperature, pressure	SVM, GBR, RF, extra trees, XGB, ANN	R^2^ > 0.99	CO_2_ uptake capacity
	Modeling and Optimizing N/O-Enriched Bio-Derived Adsorbents for CO_2_ Capture: Machine Learning and DFT Calculation Approaches	Physicochemical and structural features of biomass-based activated carbon	RBF-NN	R^2^ = 0.99, 0.974, 0.995, 0.9658, 0.9476, 0.9891 for test set predictions at (298 K and 273 K) 0.15 bar, (298 K and 273 K) 0.6 bar, and (298 K and 273 K) 1 bar, respectively	CO_2_ adsorption
